# Granisetron plus aprepitant versus granisetron in preventing nausea and vomiting during CHOP or R-CHOP regimen in malignant lymphoma: a retrospective study

**DOI:** 10.1186/s40780-019-0153-3

**Published:** 2019-11-21

**Authors:** Yoshinori Wakasugi, Satoshi Noda, Yoshihiro Ikuno, Miya Horie, Katsuyuki Kito, Hitoshi Minamiguchi, Tomohiro Terada

**Affiliations:** 1 Department of Pharmacy, Shiga University of Medical Science Hospital, 520-2192 Seta Tsukinowa-cho, Otsu-shi, Shiga Japan; 2 Department of Hematology, Shiga University of Medical Science Hospital, 520-2192 Seta Tsukinowa-cho, Otsu-shi, Shiga Japan

**Keywords:** Aprepitant, Granisetron, CHOP, Complete response, Complete protection

## Abstract

**Background:**

Cyclophosphamide, doxorubicin, vincristine, and prednisolone (CHOP) regimen includes a high dose of prednisolone (100 mg/body), which exhibits an anticancer and antiemetic effect. However, its optimal use for antiemetic therapy has not been established yet. We assessed the efficacy of granisetron plus aprepitant versus granisetron for CHOP or rituximab-CHOP (R-CHOP) regimen-induced nausea and vomiting in malignant lymphoma.

**Methods:**

This retrospective and observational clinical study included patients who received CHOP or R-CHOP regimen as initiating chemotherapy between July 2010 and March 2016 (*N* = 39). Patients were assigned to an aprepitant [aprepitant (125 mg on day 1, 80 mg on days 2–3) plus granisetron (3 mg); *n* = 15] or control regimen group [granisetron (3 mg); *n* = 24]. Complete response (CR), defined as no vomiting and no use of rescue therapy during overall phase (0–120 h), was the primary endpoint. Secondary endpoints included the time to first vomiting and using rescue medication and complete protection (CP) defined as no vomiting and no retching and/or no nausea and no rescue therapy. The patient records were investigated, and data were retrospectively analyzed.

**Results:**

CR rate CP rates did not significantly differ between the groups during the observation period (80.0% versus 83.3%, *p* = 1.000; and 80.0% versus 79.2%, *p* = 1.000, respectively). Additionally, the time to first vomiting and using rescue medication in did not significantly differ between the groups (*p* = 0.909).

**Conclusions:**

This study suggests that granisetron alone could be one treatment option in the management of CINV in patients with non-Hodgkin lymphoma receiving CHOP or R-CHOP regimen.

## Background

Chemotherapy-induced nausea and vomiting (CINV) is one of the most severe adverse events associated with cancer chemotherapy, and often affects patients’ quality of life [1, 2]. Thus, the management of CINV is crucial for successful cancer chemotherapy. The incidence of CINV is greatly influenced by the emetogenic potential of the anticancer drug. Anticancer drugs and regimens are classified into the following four categories regarding the risk of CINV: high (high-emetic chemotherapy: HEC) risk > 90%; moderate (moderate-emetic chemotherapy: MEC) risk, 30 to 90%; low (low-emetic chemotherapy: LEC) risk, 10 to 30%; and minimal risk, < 10% [3].

The cyclophosphamide, doxorubicin, vincristine, and prednisolone (CHOP) regimen is the standard chemotherapy for primary aggressive non-Hodgkin lymphoma. This regimen includes doxorubicin (50 mg/m^2^), cyclophosphamide (750 mg/m^2^), vincristine (1.4 mg/m^2^), and prednisolone (100 mg/body). The R-CHOP regimen has been shown to significantly extend overall survival compared with the CHOP regimen in patients with diffuse large B-cell lymphoma, and is regarded as a standard therapy [4].

The National Comprehensive Cancer Network (NCCN), American Society of Clinical Oncology (ASCO), and Japan Society of Clinical Oncology antiemesis guidelines classify the AC regimen (doxorubicin at 60 mg/m^2^ and cyclophosphamide at 600 mg/m^2^) as HEC, and recommend a combination of 5-HT3 receptor antagonist, dexamethasone, and NK1 receptor antagonist for HEC [5–7]. The CHOP regimen has not been categorized according to the emetogenic risk in the ASCO, NCCN, or Multinational Association of Supportive Care in Cancer/European Society of Medical Oncology guidelines. It is unclear whether AC used as a component of the CHOP regimen is also highly emetic. On the other hand, the CHOP regimen has been categorized HEC on the basis of consensus-based recommendation in Japan Society of Clinical Oncology antiemesis guidelines [5]. However, in a clinical setting, the aprepitant tend to be not used as an antiemetic agent for the management of CINV induced by CHOP regimen, because patients are administered 100 mg of prednisolone orally for 5 days, which may decrease the risk of CINV. Actually, in a recent observational study conducted in Japan, 79% of patients with hematological malignancies who received HEC, including CHOP regimen, did not used aprepitant [8]. Against this background, it is unknown whether NK1 receptor antagonists are necessary for the CHOP regimen. Therefore, we assessed the efficacy of the combination of granisetron and aprepitant for the management of CINV in Japanese patients with non-Hodgkin lymphoma receiving CHOP or R-CHOP regimen.

## Methods

### Patients

This study included patients who received CHOP or R-CHOP regimen as initial chemotherapy between July 2010 and March 2016 at Shiga University of Medical Science Hospital hematology (*N* = 39). The patients using aprepitant and granisetron as antiemetic drugs were classified into the aprepitant regimen group, whereas those using only granisetron were classified into the control regimen group. The exclusion criteria in this study included the use of palonosetron, prophylactic administration of antiemetic drugs (dopamine-2 (D_2_) receptor antagonist) and the use of methylprednisolone for the treatment of infusion reaction induced by rituximab, not receiving doxorubicin, cyclophosphamide or prednisolone.

### Therapies

The CHOP regimen consisted of cyclophosphamide (750 mg/m^2^ intravenously), doxorubicin (50 mg/m^2^ intravenously), and vincristine (1.4 mg/m^2^ intravenously), on day 1, and prednisolone (100 mg orally) on days 1–5. The day on which the administration of the antiemetic drug was started was set as day 1. In both groups, in the case of the CHOP regimen, 3 mg of granisetron was administered in 30 min, doxorubicin at 50 mg/m^2^ was administered in 30 min, followed by vincristine at 1.4 mg/m^2^ in 30 min and cyclophosphamide at 750 mg/m^2^ in 2 h on day 1. Prednisolone was administered at 100 mg daily (55 mg in the morning, 30 mg in the afternoon 15 mg in the evening) for 5 days from day 1. In the case of the R-CHOP regimen, in addition to administration of the CHOP regimen, rituximab was administered on day 0. No additional administration of corticosteroid other than CHOP was performed as a premedication of rituximab. The use of aprepitant was selected at the discretion of the doctor, and 125 mg was administered 1 h before doxorubicin on day 1, and 80 mg on each of days 2 and 3, only to the aprepitant regimen group.

### Assessments

The primary endpoints included proportions of patients with complete response (CR; no vomiting and no use of rescue therapy) in the acute phase (0–24 h), delayed phase (24–120 h), and overall phase (0–120 h). The secondary endpoints included the proportion of patients with complete protection (CP; no vomiting and no retching and/or no nausea, no use of rescue therapy) and time to first vomiting and using rescue medication. Retrospective investigations were performed using electronic medical records. Retching/nausea or vomiting was considered to have occurred if there was a description of it in the electronic medical records. The evaluation period was 120 h after the start of chemotherapy.

### Statistics

Descriptive data are expressed as mean ± SD. The Mantel–Haenszel test was used to analyze the CR rate and CP rate in the evaluation period. The Kaplan–Meier method was used to estimate the time to first onset of vomiting and using rescue medication. All comparison tests were two-sided. *p < 0.05* was considered to be statistically significant. The ethics committee of Shiga University of Medicine approved the protocol (approval number: 28–27). This study is an observational retrospective analysis. Consequently, this study is not registered and does not have a trial registration number.

## Results

### Patient baseline clinical characteristics

In this study, 58 patients who were administered CHOP or R-CHOP regimen were enrolled. Overall 19 patients were excluded, for the following reasons: nine patients used palonosetron, five had prophylactic administration of antiemetic drugs in addition to granisetron, and five did not receive doxorubicin, cyclophosphamide, or prednisolone. Overall, 24 and 15 patients in the control and aprepitant regimen group were evaluable, respectively. Table 1 shows the patient backgrounds. As shown in this table, there was no significant difference in age or relative dose intensity between the groups, but there was significant difference in sex, regimen, and type of histology.

**Table 1:** Patient backgrounds treated with CHOP or R-CHOP regimen in malignant lymphoma with or without aprepitant

	Total	Aprepitant regimen group	Control regimen group	*p* value
No. of patients	39	15	24	
Age				
Median (range)		60 (37–75)	67 (27–76)	0.71
< 50 years	6	2	4	0.063
> 50 years	33	13	20	
Sex				
Male	19	6	13	0.042
Female	20	9	11	
R-CHOP	25	8	17	0.029
CHOP	14	7	7	
Type of histology				
FL	6	3	3	0.049
DLBCL	22	7	15	0.036
Other^a^	11	5	6	0.053
RDI of CHOP (%)		94	91	0.33

Statistical analyses were performed with the Chi-squared test

(%) Relative dose intensity (RDI) = dose intensity (mg/m^2^/week)/planned dose intensity (mg/m^2^/week) × 100

*Abbreviations*: *R-CHOP* Rituximab, cyclophosphamide, doxorubicin, vincristine, prednisolone, *CHOP* Cyclophosphamide, doxorubicin, vincristine, predonisolone, *FL* Follicular lymphoma, *DLBCL* Diffuse large B-cell lymphoma

^a^*PTCL,* peripheral T cell lymphoma; *ALCL,* anaplastic large cell lymphoma, *AITL,* angioimmunoblastic T cell lymphoma; *IVL,* intravascular lymphoma

### Antiemetic effects

**Fig. 1:**
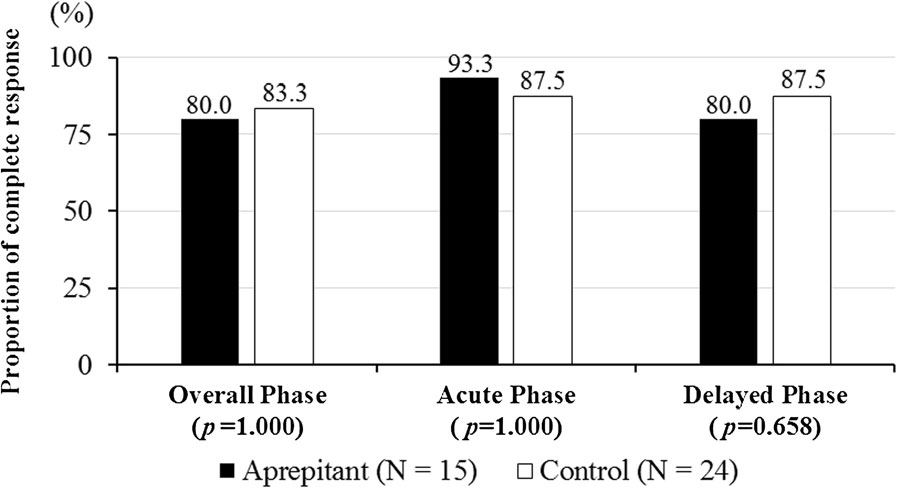
Complete response rate during the overall (0–120 h), acute (0–24 h), and delayed (24–120 h) phases. For the aprepitant regimen: *n* = 15. For the control regimen *n* = 24. *p* = 1.000, 1.000, 0.658 versus control regimen

**Fig. 2:**
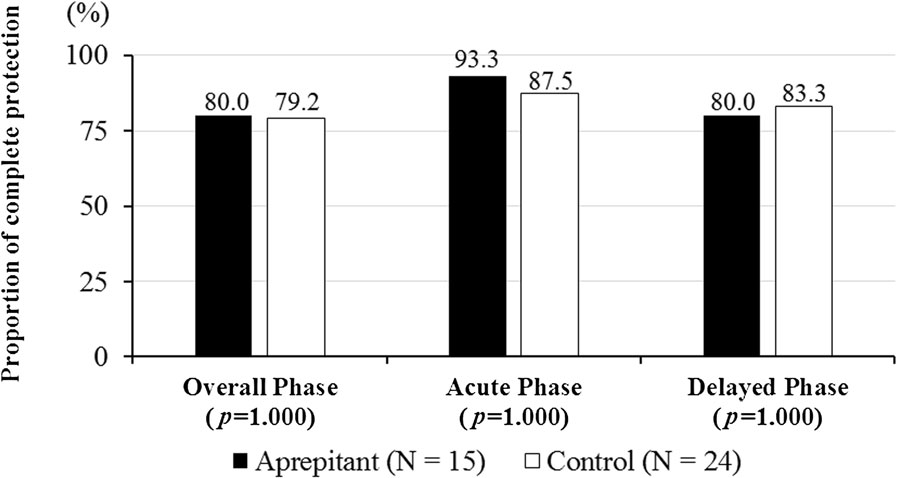
Complete protection rate during the overall (0–120 h), acute (0–24 h), and delayed (24–120 h) phases. For the aprepitant regimen: *n* = 15. For the control regimen *n* = 24. *p* = 1.000, 1.000, 1.000 versus control regimen

**Fig. 3:**
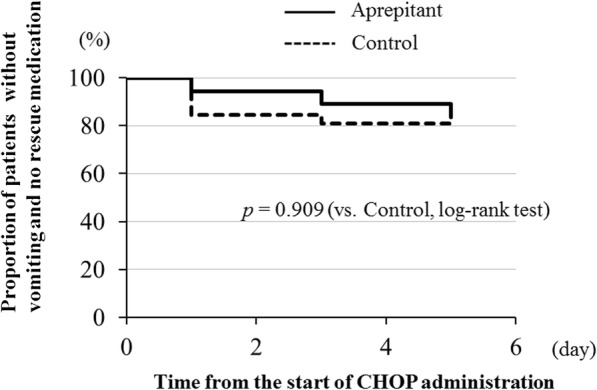
Time to first vomiting and using rescue medication during the overall (0–120 h) phases. The two groups did not show a statistically significant difference in this variable (*p* = 0.909)

Figure 1 shows the CR rates for the overall phase, acute phase, and delayed phase within the evaluation period. There were no significant differences in CR rate in the overall phase (80.0 vs. 83.3%, *p* = 1.000), acute phase (93.3 vs. 87.5%, *p* = 1.000), or delayed phase (80.0 vs. 87.5%, *p* = 0.658) between the aprepitant and control regimen groups. Figure 2 shows the CP rates for overall phase, acute phase, and delayed phase within the evaluation period. In the aprepitant and control regimen groups, there was no significant difference in CP rate in overall phase (80.0 vs. 79.2%, *p* = 1.000), acute phase (93.3 vs. 87.5%, *p* = 1.000), and delayed phase (80.0 vs. 83.3%, *p* = 1.000). The time to first vomiting and using rescue medication is shown in Fig. 3. Again, the two groups did not show a statistically significant difference in this variable (*p* = 0.909).

## Discussion

In this study, we compared antiemetic effect with granisetron and aprepitant combined with granisetron at the time of CHOP or R-CHOP regimen. We found no significant difference in CR rate, CP rate, or time to first onset of vomiting and using rescue medication in the evaluation period. CR rate and CP rate exceeded approximately 80% in the acute, delayed, and overall phases. These results suggest that granisetron only could be one treatment option in patients with non-Hodgkin lymphoma who have received CHOP or R-CHOP regimen. In a previous study, Takahashi et al. performed a retrospective study to evaluate the effects of oral and intravenous first-generation 5-HT3 receptor antagonists (ondansetron) in patients with non-Hodgkin lymphoma who received R-CHOP or CHOP regimen [9]. They reported that the CR rate did not differ between the two groups (overall: 82.1% vs. 78.8%, *p* = 0.77; acute phase: 87.2% vs. 90.9%, *p* = 0.72; delayed phase: 84.6% vs. 81.8%, *p* = 0.76), suggesting that ramosetron is one of the optimal options for preventing CINV in patients treated with CHOP or R-CHOP regimen. The use of the first-generation 5-HT3 antagonist for the MEC regimen, including AC therapy, which does not contain large amounts of steroids, unlike the CHOP regimen, has been reported with a CR rate of 69% in the acute phase and 49% in the delayed phase [10]. Our results included a higher rate of acute CR (93.3%) than in that previous report. This discrepancy appears to be largely due to the antiemetic effect caused by the use of a high dose of prednisolone. Oral administration of prednisolone to days 4 to 5 of the CHOP regimen may reduce or delay vomiting, even without aprepitant.

Morita et al. performed a prospective study to evaluate the efficacy of aprepitant for patients with non-Hodgkin lymphoma who experienced nausea, vomiting, or anorexia exceeding grade 1 in the first course of CHOP regimen and who received aprepitant for 3 days in addition to granisetron with the second course. With the second course, the number and severity of CINV episodes decreased compared with those in the first course. Nausea and anorexia were also significantly reduced (*p* < 0.05) [11]. The authors reported that the addition of aprepitant to 5-HT3 receptor antagonist appears to be effective for CINV or anorexia in patients who have received CHOP regimen. However, they only reported an analysis of the effect of aprepitant on patients in whom granisetron treatment as an antiemetic therapy had failed. Therefore, it is unknown which is better: granisetron or aprepitant + granisetron for CHOP during the first regimen. Zeng et al. also performed a prospective investigation of the efficacy and safety of triple therapy with aprepitant, ondansetron, and prednisolone in patients with non-Hodgkin lymphoma receiving rituximab + cyclophosphamide + epirubicin + vincristine + prednisolone (R-CEOP) or CEOP regimen [12]. They concluded that the CR rate upon triple therapy was statistically superior to the double therapy (ondansetron and prednisolone) in the overall observation period (76.5% vs. 56.0%; *p* = 0.03). However, no comparison of the emetogenic intensity between CHOP and CEOP has been performed; it is not possible to conclude that aprepitant+first-generation 5-HT3 antagonist are superior to the first-generation 5-HT3 antagonist in preventing CHOP regimen-induced acute and delayed emesis.

Because aprepitant has CYP3A4 inhibitory activity, attention must be paid to drug interaction when it is used in combination with CYP3A4 substrates. It has been reported that the frequency of occurrence of chemotherapy-induced peripheral neuropathy due to vincristine, a CYP3A4 substrate, increased when aprepitant was used in combination with the CHOP regimen, and it is possible that aprepitant may contribute to an increase of vincristine-induced side effects [13]. Considering that high antiemetic rates can be expected without using aprepitant and that vincristine interact with CYP3A4, aprepitant might not be needed in patients with non-Hodgkin lymphoma receiving CHOP or R-CHOP regimen. In the present study, the frequency of peripheral neuropathy was no significant difference between the aprepitant regimen group and the control during the first course (7.1% vs 5.0%; *p* = 0.11).

However this our exploratory analysis focus on short-term peripheral neuropathy. Therefore, further long-term, prospective studies are needed to assess chemotherapy-induced peripheral neuropathy in patients undergoing treatment with CHOP or R-CHOP regimen in combination with aprepitant.

This study had some limitations, such as its retrospective nature and inclusion of a small number of patients from a heterogeneous population. Additionally, the presented study only evaluated the period of 0–120 h during the first cycle. A previous study reported that several patients experienced vomiting during 120–168 h after CHOP chemotherapy [14].

In the present study, there are significant differences in the number of female patients between the aprepitant regimen group and the control regimen group. Female is reported to be a risk factor of emesis induced by chemotherapy [15]. In the present study, no difference between granisetron plus aprepitant and granisetron was observed in CR or CP in female patients with treated CHOP or R-CHOP regimen (CR: 77.8% vs 72.7%; *p* = 1.00; CP: 77.8% vs 81.8%; *p* = 1.00). Although not significant, granisetron plus aprepitant tend to be high CR for female patient receiving CHOP or R-CHOP regimen. Recently, Yoshida et al. reported that female gender and young age were risk factors for early-phase nausea, while female gender remained a risk factor for late-phase CINV in patients with haematological malignancies. They also reported that CR and complete control were, not significantly, increased by 8.6 and 13.9%, respectively, in patients receiving triple antiemetics (aprepitant+ 5-HT3 receptor antagonist +dexamethasone) in CHOP-like regimens, compared to those with double antiemetics (5-HT3 receptor antagonist +dexamethasone) [16]. Considering the result of above study, aprepitant might be considered for HEC, especially in young female with non-Hodgkin lymphoma receiving CHOP or R-CHOP. However, owing to the small number of patients enrolled in the study and the exploratory nature of the analysis, no conclusions could be drawn. Therefore, further large-scale, prospective studies are necessary to appropriately prevent CINV in those undergoing treatment with CHOP regimen.

## Conclusion

The results of this study suggest that granisetron alone could be one treatment option in the management of CINV in patients with non-Hodgkin lymphoma receiving CHOP or R-CHOP regimen. However, this study had certain limitations and further work on this issue is necessary.

## Data Availability

The datasets generated and/or analyzed during the current study are not publicly available because transfer of data containing personal information outside Shiga University of Medical Science Hospital is not allowed.

## References

[1] Ballatori E, Roila F (2003). Impact of nausea and vomiting on quality of life in cancer patients during chemotherapy. Health Qual Life Outcomes.

[2] Grunberg SM, Boutin N (1996). Impact of nausea/vomiting on quality of life as a visual analogue scale-derived utility score. Support Care Cancer.

[3] Hesketh PJ, Kris MG (2017). Antiemetics: American Society of Clinical Oncology clinical practice guideline update. J Clin Oncol.

[4] Coiffier B, Lepage E (2002). CHOP chemotherapy plus rituximab compared with CHOP alone in elderly patients with diffuse large-B-cell lymphoma. N Engl J Med.

[5] Japan Society of Clinical Oncology. Guidelines for appropriate use of antiemetic drugs (version 2.2, 2015). Available at http://jsco-cpg.jp/item/29/index.html. Accessed 12 Mar 2019.

[6] National Comprehensive Cancer Network. Clinical practice guidelines in oncology: Antiemesis (Ver1, 2019). Available at https://www.nccn.org/professionals/physician_gls/pdf/antiemesis.pdf. Accessed 12 Mar 2019.

[7] MASCC/ESMO Antiemesis Guidline 2016. Available at https://www.mascc.org/asset/Guidlines-Tools/mascc_antiemetic_guidlines_establish_2016_v.1.2.pdf. Accessed 12 Mar 2019.

[8] Tamura K, Aiba K (2015). Testing the effectiveness of antiemetic guidelines: results of a prospective registry by the CINV study Group of Japan. Int J Clin Oncol.

[9] Takahashi T, Kumanomidou S (2016). A retrospective study of R-CHOP/CHOP therapy-induced nausea and vomiting in non-Hodgkin's lymphoma patients: a comparison of intravenous and oral 5-HT3 receptor antagonists. Int J Hematol.

[10] Warr DG, Hesketh PJ (2005). Efficacy and tolerability of aprepitant for the prevention of chemotherapy-induced nausea and vomiting in patients with breast cancer after moderately emetogenic chemotherapy. J Clin Oncol.

[11] Morita M, Kishi S (2017). Efficacy of aprepitant for CHOP chemotherapy-induced nausea, vomiting, and anorexia. Curr Probl Cancer.

[12] Song Z, Wang H (2017). Efficacy and safety of triple therapy with aprepitant, ondansetron, and prednisone for preventing nausea and vomiting induced by R-CEOP or CEOP chemotherapy regimen for non-Hodgkin lymphoma: a phase 2 open-label, randomized comparative trial. Leuk Lymphoma.

[13] Okada N, Hanafusa T (2014). Risk factors for early-onset peripheral neuropathy caused by vincristine in patients with a first administration of R-CHOP or R-CHOP-like chemotherapy. J Clin Med Res.

[14] Miyata Y, Yakushijin K (2016). A prospective study of the antiemetic effect of palonosetron in malignant lymphoma patients treated with the CHOP regimen. Int J Hematol.

[15] Warr D (2014). Prognostic factors for chemotherapy induced nausea and vomiting. Eur J Pharmacol.

[16] Yoshida I, Tamura K (2019). Prophylactic Antiemetics for Haematological malignancies: prospective Nationwide survey subset analysis in Japan. In Vivo.

